# Supplementary material for: A set of multi-entry identification keys to African frugivorous flies (Diptera, Tephritidae)
Source: Zookeys. 2014 Jul 24;(428):97–108. doi: 10.3897/zookeys.428.7366 (PMC4143993; doi:10.3897/zookeys.428.7366)
Supplement: Supplementary material 6 — Key to Ceratitis [file zookeys-428-097-s006.zip › SF6_ZooKeys_key to Ceratitis/key/SF6_ZooKeys_key to Ceratitis/Media/Html/Ceratitis cristata.htm]

***Ceratitis*** ***cristata*** **(Bezzi)**

*Hoplolopha* *cristata* Bezzi, 1920: 234.

Body length: 6.25 (5.70-6.70) mm; wing length: 6.75 (5.80-7.70)
mm.

Male

Head. Antenna yellow or yellow-orange.
First flagellomere three times as long as pedicel. Arista with short hairs.
Frons convex, in lateral view not projecting at antennal implant; yellowish
area in between orbital bristles silvery shining; with short scattered hairs
which are distinctly darker than frons. Two black frontal bristles well-developed.
Three black orbital bristles, slightly elevated (�cristate�); basally slightly
flattened laterally, sometimes truncated in lower part. Face yellow-white;
lower eye margin silvery pilose, posteriorly with small, darker patch. Genal
bristle long and dark; genal setulae dark, well-developed.

Thorax.
Postpronotum white, without spot. Scutum, ground colour orange-grey, with
streaks and darker markings but no distinct spots or clearly defined stripes.
Scapular setae dark. Scutellum yellowish white, basally without distinct basal
spots, but with ill defined basal patches, sometimes missing; apically with
three black separate spots, reaching to basal third or midlength. Subscutellum
with three spots, merging dorsally; completely black in some cases.
Anepisternum white, lower margin narrowly yellow; one black anepisternal
bristle, otherwise pale pilosity except for few dark hairs along upper and
lower margin.

Legs. Greyish white or more yellowish; elongated, stilt-like; no
distinct bushy or feathery pilosity. Fore femur posterodorsal row pale, apically with a
few darker hairs, sometimes completely dark; ventral
bristles black; hind femur sometimes with dispersed black bristles
anteroventrally and posteroventrally. Otherwise legs with mixed pale and
darkish pilosity.

Wing. Banding, yellow-brown markings. Cubital band free; medial
band joining marginal band, but usually with narrow bridge. Crossvein r-m at
basal 2/5 to third of discal cell. Vein R_1_ extending beyond
crossvein r-m. Crossvein dm-cu variable.

Abdomen. Yellow to orange. Tergite 1 with posterior margin very
narrowly brown. Tergite 2 with posterior margin greyish; tergite 4 wholly grey
except anterior margin. Tergite 3 brown-black except for posterior margin.

Female

As male, except for the following
characters. Orbital bristles not cristate, sometimes only two present, even
asymmetrically in one specimen; frons with median part sometimes less silvery. Oviscape
shorter than preabdomen.

(Redescription after De Meyer &
Copeland, 2001)
